# Evaluating the efficiency and ergonomics of a novel smart surgical lighting system: a passive oddball experiment with EEG measurements to assess workplace strain in clinical settings

**DOI:** 10.3389/fmedt.2025.1584606

**Published:** 2025-09-16

**Authors:** Tim Schneider, Dirk Weyhe, Merle Schlender, Timur Cetin, Navid Tabriz, Verena Uslar

**Affiliations:** Department for Human Medicine, University Clinic for Visceral Surgery, Pius-Hospital Oldenburg, University Medicine Oldenburg, Oldenburg, Germany

**Keywords:** ergonomics in surgery, event-related potentials, NASA-TLX, neuroergonomics, P300, smart surgical lighting, workplace strain

## Abstract

**Introduction:**

The primary objective of this study was to evaluate the efficiency and ergonomic benefits of a novel surgical lighting system developed within the *SmartOT* project. The developed system aims to automatically prevent shadows on the surgical field, eliminating the need for frequent manual adjustments, which is common with conventional surgical lights. Additionally, the study seeks to explore the feasibility of using EEG recordings as an objective method for assessing workplace strain in clinical settings, thereby laying the groundwork for future studies focused on reducing the workload of medical personnel.

**Methods:**

To achieve these objectives, we conducted a passive Oddball experiment involving EEG measurements to assess the impact of the new lighting system on workplace strain. Participants performed a task requiring them to identify specific LEGO® pieces. The study involved 30 participants (13 females, 17 males), with errors being tracked as an additional measure of cognitive load. The experimental setup was informed by previous research, which established a method for objectively determining workload generated by AR and VR technologies in clinical settings. In that research, EEG signals were recorded during surgical planning under different conditions, revealing trends in cognitive load and validating the utility of EEG for workload assessment.

**Results:**

The NASA Task Load Index (NASA-TLX) analysis revealed significantly lower mental demand, temporal demand, effort, and frustration scores for the smart surgical lamp compared to the manual lamp conditions, with mandatory and optional adjustments. However, there were no significant differences between the smart and conventional lamp in the dimensions of physical demand and performance. Similarly, EEG recordings indicated a higher P300 amplitude at electrode Fz following the smart lamp condition (*p* = 0.037), reflecting less cognitive load; latencies did not differ between conditions. Error analysis confirmed fewer errors and shorter processing times for the smart lamp.

**Conclusions:**

The measurements of NASA-TLX and EEG after running simulated surgical tasks showed that the *SmartOT* prototype significantly reduced errors and workload compared to the conventional surgical lamp. These findings reflect the capability of smart surgical lighting in improving patient safety and efficiency within operating theaters.

## 1 Introduction

Good lighting is essential in the operating room, as it plays a key role in ensuring precision and safety during surgeries (1). Modern surgical lights are designed to offer a clear, shadow-free view of the surgical site, enabling surgeons to carry out complex procedures with accuracy. However, despite advancements in lighting technology, current systems still have notable limitations that can impact their effectiveness and the overall efficiency of surgeries (2).

A common challenge with traditional surgical lighting is the frequent shadowing caused by staff or instruments blocking the light source. These shadows can make it harder for surgeons to see vital anatomical details, increasing the risk of mistakes. To address this, surgical teams often have to adjust the lights multiple times during a procedure, which is both time-consuming and disruptive (2). Constantly modify the lighting not only slows down the workflow but also adds physical strain on the team, who must repeatedly reposition the lights to ensure clear visibility.

Traditional surgical lights pose an additional critical challenge: the risk of contamination. Since surgical light handles are often touched and adjusted during procedures, they can become a source of bacterial transmission within the sterile field. Schweitzer et al. pointed out that these handles are potential carriers of pathogens, which can increase the risk of infections (3). This highlights the importance of developing lighting systems that reduce or eliminate the need for manual contact, thereby helping to minimize the danger of cross-contamination.

Although, as described by Sharma et al., surgical lighting has significantly improved over the past 50 years, certain issues cannot be resolved using conventional surgical lighting systems (SLS) (4). Knust et al. report that during surgeries using conventional SLS, the lighting needs to be readjusted every 7.5 min to maintain adequate illumination of the surgical field (2). These adjustments resulted in interruptions to the surgical procedure in 64% of cases, unavoidably extending surgery duration and increasing associated costs. These drawbacks underline the need for an automatic lighting system to address these issues effectively.

In response to these issues, the *SmartOT* project has developed an innovative surgical lighting system designed to address the limitations of conventional SLS (5). This new system employs a network of ceiling-mounted lighting modules that automatically adjust to prevent shadows from forming on the surgical field. By eliminating the need for manual repositioning, the *SmartOT* system not only enhances visibility but also reduces the ergonomic strain on the surgical team and lowers the risk of contamination.

The introduction of advanced surgical lighting systems like *SmartOT* also raises the question of how these technologies impact the cognitive load and workplace strain experienced by medical professionals. Surgical procedures are mentally and physically demanding, and excessive cognitive load can potentially have negative impact on the surgeons performance (6). Therefore, evaluating the ergonomic and cognitive impacts of new surgical technologies is crucial for ensuring that they not only improve surgical outcomes but also support the well-being of the healthcare workers who use them.

To investigate the cognitive load of users of such an innovative lighting concept and assess whether autonomous operating room (OR) lighting can reduce the workload. Cognitive load can either be evaluated subjectively through questionnaires administered after usage, or objectively during usage using biosignals measurement methods (7). From a neuroergonomic perspective, objective measurement methods should be prioritized over subjective questionnaires, as the latter rely on self-reports that are prone to various biases. Objective measurements provide more accurate and reliable data by directly and objectively capturing neurophysiological and behavioral responses in real-time through biosignals (8). According to Dias et al., an optimal approach for assessing surgeons' cognition involves a combination of subjective and objective measurement methods to evaluate cognitive load effectively (7).

To objectively assess the impact of the *SmartOT* lighting system, this study employs a passive Oddball experiment with EEG measurements, which has proven to be a valuable tool for determining objective workload in a previous study (9). This method allows for the objective monitoring of cognitive load by analyzing brainwave patterns, providing an objective measure of workplace strain.

Objective cognitive workload is measured via Event-Related Potentials (ERP), whereas subjective ratings are assessed via the NASA-Task Load Index (NASA-TLX) questionnaire. This study combined physiological and behavioural measures as an attempt toward comprehensive investigation of how smart lighting systems impact end-users in operating theatres. The hypotheses of this study are therefore:

• *Hypothesis1:* Smart surgical lighting will reduce cognitive workload, as expressed by higher ERP amplitudes in relevant ERP-components (p300) and lower scores in NASA-TLX questionnaire, compared to manually controlled SLS.
• *Hypothesis 2:* The smart lighting system will reduce the error rate in comparison to manually controlled SLS.
• *Hypothesis 3:* Participants will be faster when using the smart lighting in comparison to when using the normal SLS.

The study aims to assess the innovative *SmartOT* lighting system's ability to enhance operating room efficiency and ergonomics by reducing shadow-related disruptions. Through the automatic optimization of lighting, the system aims to reduce manual interventions and mental workload. The study also investigates the potential of EEG recordings as a robust measure for objectively quantifying workplace strain, giving insights into reducing the burden on medical personnel.

## 2 Material and methods

### 2.1 Participants

All participants provided their written informed consent prior to the start of the study. Ethical approval was obtained from the Medical Ethics Committee (24.05.2023, reference number: 2023-126) as well as from the Data Protection and Information Security Management Office at the University of Oldenburg. The study adhered to the principles outlined in the Declaration of Helsinki and complied with Good Clinical Practice guidelines. To keep privacy, all data were pseudonymized, with each participant identified only by an exclusive numerical code.

A total of 32 right-handed participants were initially recruited. Due to poor data quality caused by unstable Bluetooth connection of the mobile EEG, two participants had to be excluded retrospectively. The following analyses refer to the remaining 30 participants (13 females, 17 males) with a mean age of 24.9 years (SD = 4.33). The participants were students with no practical experience in surgical operations. Inclusion criteria included being of legal age and right-handedness. Exclusion criteria included fatigue, excessive caffeine consumption, the use of psychoactive substances, and neurological impairments. The exclusion criteria for the study were assessed prior to the experiment using a medical history questionnaire. Based on the responses, none of the participants met the exclusion criteria, and all were deemed eligible for inclusion in the study. Participants were recruited via the university's internal learning management system, which serves as a centralized platform for communication and course coordination.

### 2.2 Stimuli

Two sinusoidal tone pips served as task-irrelevant auditory stimuli: a standard tone with a frequency of 1,200 Hz and a target tone with a frequency of 2,000 Hz, both presented at a sound level of 72 dB SPL. The speakers were positioned 1.5 meter from the participants. The tones were delivered to participants for 100 ms via two loudspeakers (iLoud MTM, IK Multimedia) connected to an amplifier (the t.amp E4-130, Thomann). To prevent speaker popping, the tones included a rise/fall time of 10 ms. The interstimulus interval was set at 1 s, with standard tones presented at an 80% probability and target tones at a 20% probability. The presentation was controlled to ensure that target tones were not immediately followed by another target tone.

### 2.3 *SmartOT*-lighting-system

**Figure 1:**
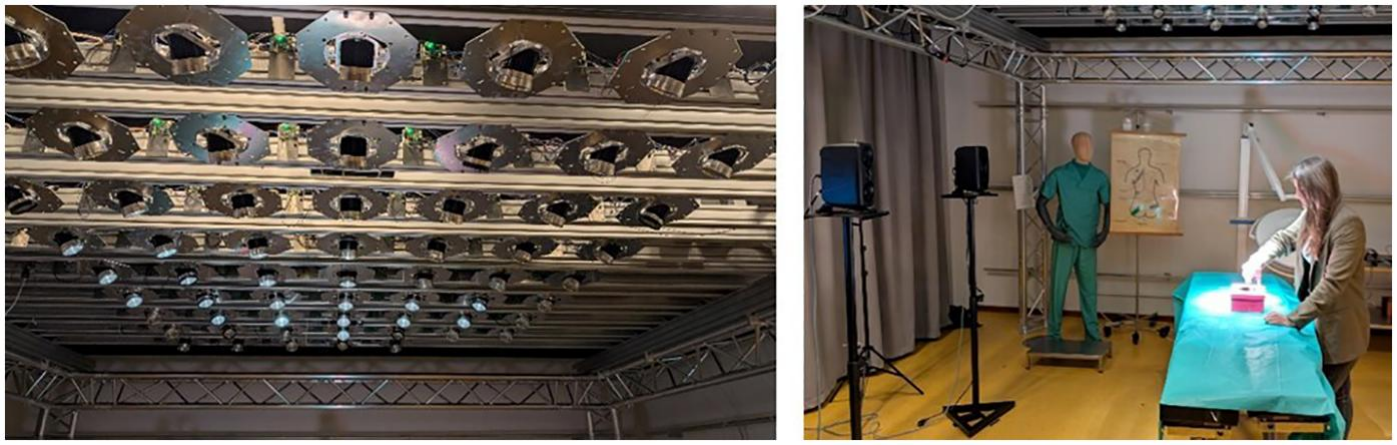
Image of the *SmartOT* prototype (left) and the general setup (right).

The *SmartOT* project is an initiative funded by the German Federal Ministry of Education and Research (File no. 13GW0264C) focused on developing a smart surgical lighting system consisting of multiple light modules (56 in total) that are autonomously controlled (Figure 1). The autonomous control is based on novel algorithms that utilize data from multiple depth cameras to detect the location of objects or individuals within the operating room that might cast shadows on the surgical site (10). Using these algorithms, light modules that would create shadows on the surgical site can be deactivated, while other modules that would not cause shadows can be activated to ensure adequate illumination of the surgical site. The control of the individual light modules operates entirely autonomously, eliminating the need for manual adjustment by the surgical team.

The system has been tested both in a simulated virtual reality setting (11) and in real-life conditions using a fully functional demonstrator (5), which is also utilized in this study.

### 2.4 Setup and procedure

As an objective measurement method, this study utilizes EEG recordings, a standardized approach to objectively assess cognitive workload (12, 13). For EEG recordings, a mobile EEG system (Smarting mobile, mbt) with 24 electrodes was used. The recorded signals were transmitted via Bluetooth to a standard desktop computer running the Windows 10 operating system (Microsoft) and sampled at a frequency of 500 Hz, saved as xdf files.

Two speakers presenting auditory stimuli were positioned 1.5 m in front of the participants. Participants stood centrally under the *SmartOT* lamp in front of a patient table. Shorter participants stood on a step to adjust for height differences. Participants remained stationary in all three conditions. In the two conditions with the manual surgical lamp, it was positioned opposite the participants. The smart lamp, mounted on a high frame, remained in the same position across all conditions, ensuring it did not interfere with the functionality of the manual lamp. The experimenter stood opposite the participants, between the speakers, ensuring they were not obstructed. The rest of the room was only dimly lit.

At the start of the session, following the participants' written consent, the EEG cap was applied. To maintain electrode impedances below 10 kΩ, 70% rubbing alcohol and abrasive conductive gel were used. Once the impedances were verified and deemed acceptable using the Smarting Streamer software (smarting streamer V 3.4.2, mbt), the experiment began.

Participants underwent three conditions in a randomized order. The order of experimental conditions was randomized individually for each participant using a MATLAB script. In all conditions, the task remained consistent. Participants were instructed to retrieve LEGO® bricks matching those in a model of seven bricks presented to them. This task involved 12 rounds per condition, with a new LEGO® model introduced in each round. Thus, participants were exposed to 12 unique models per condition, presented in random order. The box from which bricks were retrieved contained numerous similar LEGO® bricks in shape and color, increasing the task's difficulty.

Upon selecting a matching brick, participants used surgical tools to place it in a separate box with a small opening, ensuring they could no longer view or access the bricks once deposited. This required them to remember which bricks they had already placed in the box.

In each condition and during every trial, that is, once per LEGO® model, the experimenter used a dummy to cast a shadow onto the surgical site at a random time. The three conditions differed solely in the manner in which the shadow was handled. The three conditions were:

1. **Condition 1 (Smart)**: A smart surgical lamp developed in the *SmartOT* project automatically compensated for the shadow. The participants were not required to adjust the lamp by their own.
2. **Condition 2 (Mandatory Adjustment)**: Participants used a standard manually operated surgical lamp. Whenever a shadow appeared, participants were required to interrupt the task immediately and adjust the lamp to eliminate the shadow.
3. **Condition 3 (Optional Adjustment)**: Participants also used a standard surgical lamp but were only required to adjust it if they deemed it necessary. They could either eliminate the shadow or continue working in the partially shadowed field.

Before completing the manual lighting conditions, participants practiced handling the surgical lamp and were instructed to adjust it toward predefined positions to ensure familiarity with the adjustment process.

This setup allowed for the evaluation of different lighting strategies and their impact on task performance under simulated surgical conditions. The task was designed to simulate key aspects of surgical activity, including fine motor control, spatial reasoning, and working memory. Identifying and manipulating visually similar LEGO® pieces under time constraints reflects the cognitive and sensorimotor demands of intraoperative decision-making and precision-based tasks.

After participants selected seven bricks and placed them in the second box, the trial was briefly paused. The experimenter reviewed the bricks, noted errors, and returned the selected bricks to the collection to ensure the same level of difficulty across all models. The next model was then introduced. After completing all 12 models for a given condition, the condition was switched. The time required to complete each model was recorded. Errors were defined as selecting a LEGO brick with an incorrect color or shape, or omitting the brick entirely.

**Figure 2:**
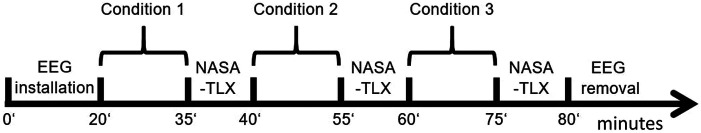
Timeline of the oddball-experiment. Conditions were presented in randomized order. The displayed times are the estimated minutes.

At the end of each condition, participants completed the NASA-TLX questionnaire to assess subjective workload. The complete experimental procedure is showed in Figure 2.

### 2.5 NASA-TLX questionnaire

The NASA-TLX was originally developed by the National Aeronautics and Space Administration for the aviation industry and has proven to be a robust tool for assessing workload across diverse settings (14). It is widely employed in studies examining the demands of complex cognitive and physical tasks (15). In the medical field, the questionnaire is frequently utilized to evaluate the workload of surgeons, nursing staff, and emergency medicine practitioners, where high mental and physical demands are typical (16, 17). Its adaptability allows for modifications tailored to specific needs and work contexts, further demonstrating its broad applicability (14).

The NASA-TLX assesses six dimensions of workload: mental demand, physical demand, temporal demand, effort, frustration, and perceived performance (15). Each dimension is rated on a scale from 0 (low) to 100 (high) by the participants, providing a comprehensive overview of subjective workload (18).

### 2.6 Data analysis and statistics

As one of the most commonly used ERP components in relation to cognitive load, this study also focuses on the analysis of the P300 component. As stated by Yu et al., this component is the most frequently used ERP component, providing robust results (19). The analysis procedure was adapted following the methodology outlined by Grassini et al. (20).

For the analysis of the P300, several preprocessing steps were performed in advance using a MATLAB script (Version: R2022b; The MathWorks Inc.) and the EEGLAB toolbox (Version: 2024.2.0). First, the data were filtered with a 0.1 Hz high-pass filter and a 40 Hz low-pass filter (Hamming windowed sinc FIR filter). Subsequently, the data were down-sampled to 256 Hz.

To mitigate potential EEG artifacts such as movement-related noise, the ICLabel plugin was used to classify independent components into categories such as brain activity, eye movements, and muscle activity. Components classified as artifacts (e.g., eye movements or muscle activity) were removed. Additionally, an automated artifact rejection procedure was applied to exclude epochs exhibiting voltage fluctuations exceeding ±100 µV or showing abnormal statistical deviations, ensuring that only clean, artifact-free epochs were included in the analysis.

Data were re-referenced to an average, and epochs were created around the “target” event (−200 to 800 ms). Baseline correction (−100 to 0 ms) was applied. Participant-specific ERPs were calculated by averaging artifact-free epochs. An automated artifact rejection procedure was applied to identify and exclude epochs containing excessive noise or extreme amplitude fluctuations. Parameters included thresholds for voltage and statistical deviation. For each participant, the ERP was calculated by averaging the cleaned, artifact-free epochs for the “target” event. Grand averages were subsequently computed across all.

All statistical analyses were conducted using repeated measures analysis of variance (ANOVA) to evaluate differences across experimental conditions. To account for violations of the sphericity assumption, Greenhouse-Geisser corrections were applied when necessary. *Post-hoc* comparisons were performed using Bonferroni correction to control for multiple comparisons. This approach was applied to subjective workload ratings derived from the NASA Task Load Index and objective cognitive workload, as measured by P300 amplitude and latency from EEG data at electrodes Fz, Cz, and Pz. For task performance evaluation, the total number of task-related errors per participant across all trials and conditions was calculated and analyzed using repeated measures ANOVA with Greenhouse-Geisser correction and Bonferroni-adjusted *post-hoc* tests. Processing time per condition was also evaluated using this approach. The alpha level was set to.05 for all statistical tests. All assumptions for repeated measures ANOVA were checked, including normality of the residuals and outlier analysis.

All statistical analyses were performed using IBM SPSS Statistics for Windows, Version 30.0.0.0 (171) (IBM Corp., Armonk, NY, USA).

## 3 Results

### 3.1 Subjective workload

The analysis of the NASA TLX questionnaire revealed the lowest “mental demand” score for the condition in which the smart surgical lamp was used (mean: 42.3; SD: 19.9). In contrast, the conditions involving the conventional SLS resulted in a mean NASA-TLX score for mental demand of 56.8 (SD: 21.2) for mandatory adjustment and 52.2 (SD: 19.9) for optional adjustment.

A repeated measures ANOVA with a Greenhouse-Geisser correction determined that the scores of the NASA-TLX showed a statistically significant difference between conditions [*F*(3.078, 89.267) = 6.86, *p* < .001]. Both values (mandatory adjustment and optional adjustment) were significantly higher than the score for the smart lamp condition (*p* = 0.017 and *p* = 0.049, respectively).

The assessment of “temporal demand” using the NASA TLX also showed the lowest values for the smart surgical lamp (mean: 30.3; SD: 18.8) compared to the mandatory adjustment (mean: 44; SD: 22) and the optional adjustment (mean: 41.8; SD: 18.7). Significant differences were observed between the smart lamp condition and the manual lamp conditions with mandatory adjustment (*p* < 0.001) and optional adjustment (*p* = 0.029).

The results for “effort” revealed a significantly lower value (*p* < 0.001) for the condition with the smart surgical lamp (mean: 36.1; SD: 17.3) compared to the manual lamp with mandatory adjustment (mean: 49.5; SD: 21.2). The detailed results of all significant comparisons from the *post hoc* test are presented in Table 1.

**Table 1:** Pairwise comparisons, based on estimated marginal means.

Pairwise comparisons							
NASA-TLX	Conditions		*M*_Diff_	Std.-error	Sig.^a^	95% confidence interval for difference^a^	
						Lower bound	Upper bound
Mental demand	Smart	Mandatory	9.667*	3.242	0.017	1.428	17.905
	Smart	Optional	12.000*	4.701	0.049	0.054	23.946
Physical demand	Mandatory	Optional	9.667*	3.619	0.037	0.470	18.863
Temporal demand	Smart	Mandatory	14.000*	3.273	0.001	5.684	22.316
	Smart	Optional	12.833*	4.622	0.029	1.090	24.577
Effort	Smart	Mandatory	13.833*	3.336	0.001	5.356	22.311
	Smart	Optional	10.333	4.207	0.061	−0.356	21.022
Frustration	Smart	Mandatory	−13.667*	4.004	0.006	−23.840	−3.493
	Smart	Optional	−8.333*	3.054	0.032	−16.094	−0.572

* The mean difference is significant at the .05 level.

^a^ Adjustment for multiple comparisons: Bonferroni.

**Figure 3:**
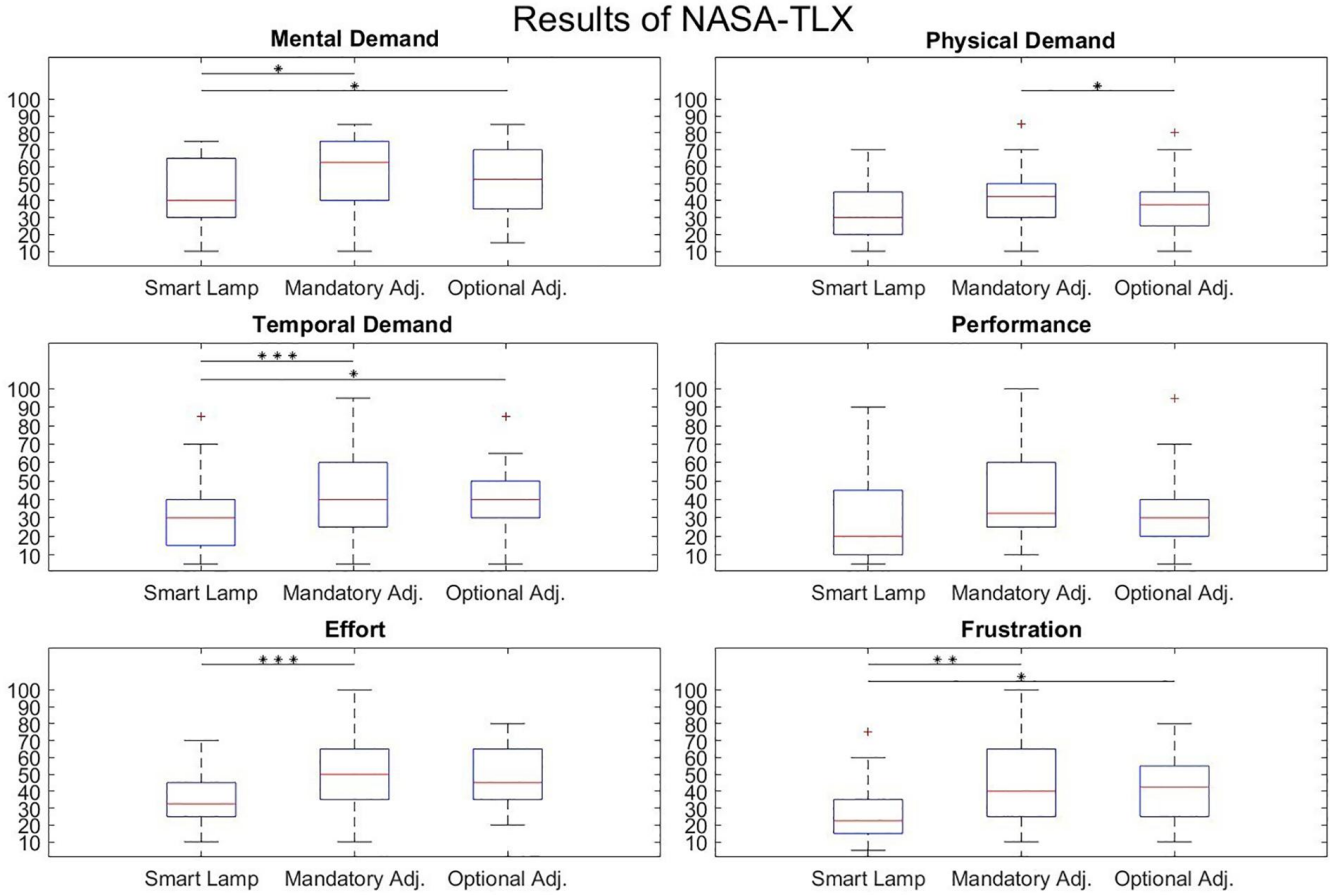
NASA-TLX scores for smart surgical lamp and traditional surgical lamp with mandatory and optimal adjustment. The central line represents the median, while the lower and upper edges of the box correspond to the 25th and 75th percentiles, respectively. The asterisks represent the level of significance (*p < 0.05; **p < 0.01; ***p < 0.001).

The analysis of the NASA-TLX questionnaires for subjectively perceived frustration also showed the lowest score for the smart surgical lamp (mean: 26.5; SD: 17.1). The smart lamp demonstrated a significantly lower score (*p* = 0.032) compared to the traditional surgical lamp with optional adjustment (mean: 45.6; SD: 23.6) and a significant lower score (*p* = 0.006) compared to the traditional surgical lamp with mandatory adjustment (mean: 42.5; SD: 19.2). Boxplots for all categories of the NASA-TLX questionnaire are presented in Figure 3.

### 3.2 Electrophysiological recordings

**Figure 4:**
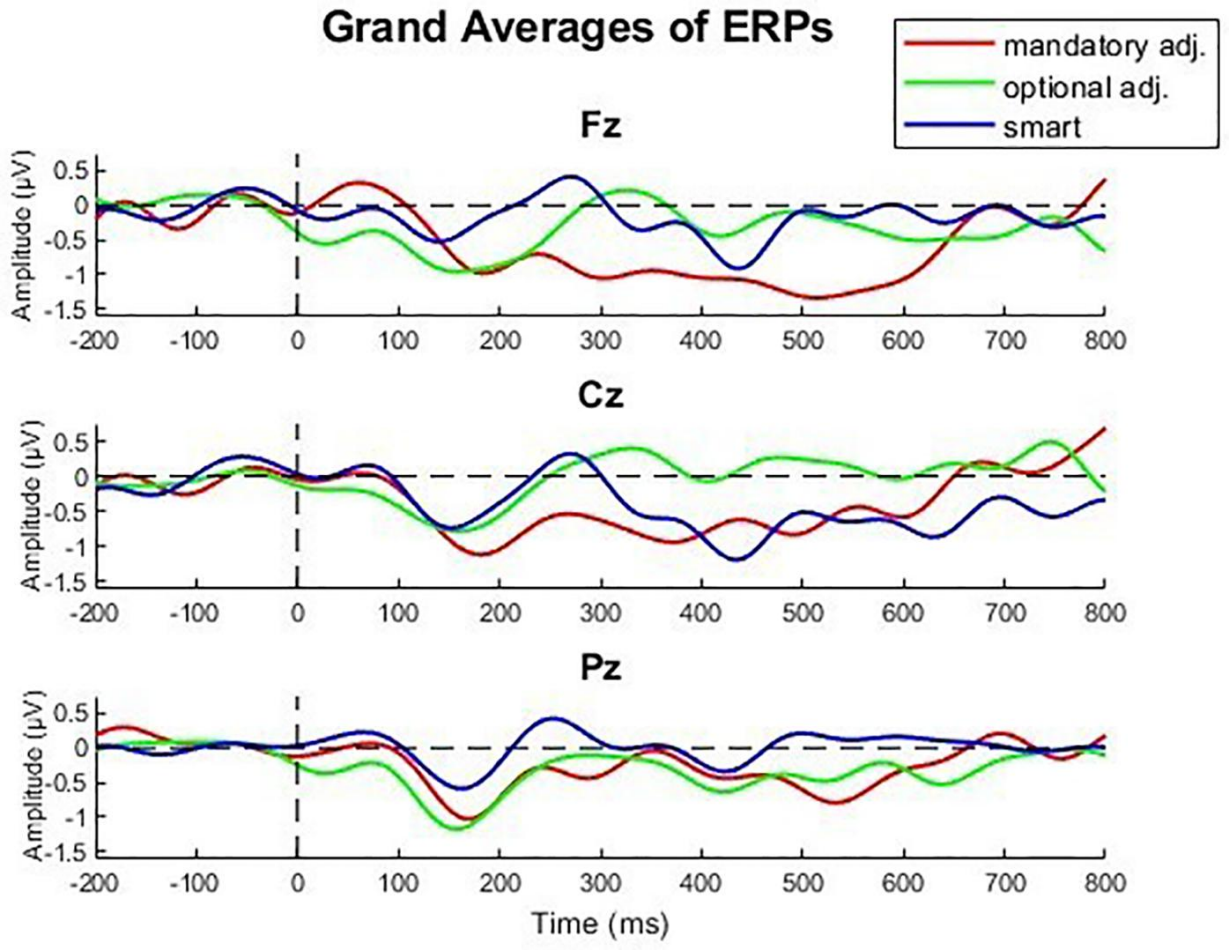
Grand averages of the central electrodes Fz, Cz, Pz over all participants.

To objectively assess the participants' cognitive load, the EEG data were analyzed with a focus on the P300 component. The preprocessing steps described in Chapter 2.6 were applied beforehand. The resulting grand averages for the electrodes Fz, Cz, and Pz are presented in Figure 4.

A repeated measures ANOVA with a Greenhouse-Geisser correction determined that the **amplitudes** of the P300 showed a statistically significant difference between conditions [*F*(1.66, 41.42) = 4.82, *p* = .018, *η*^2^ = 0.162]. A Bonferroni-corrected *post-hoc* test revealed a higher P300 amplitude in the Fz electrode (*p* = .011) when using the smart lamp compared to the manual lamp (mandatory adjustment) [*M*_Diff_ = 1.503, 95% CI (.301, 2.706)].

Regarding the **latencies** of the observed P300 components the repeated measures ANOVA with a Greenhouse-Geisser correction determined no statistically significant difference between conditions [*F*(1.948, 52.59) = 1.2, *p* = .310, *η*^2^ = 0.042]. All P300 component latencies occurred between 282 and 316 ms following stimulus onset.

### 3.3 Errors and processing time

**Figure 5:**
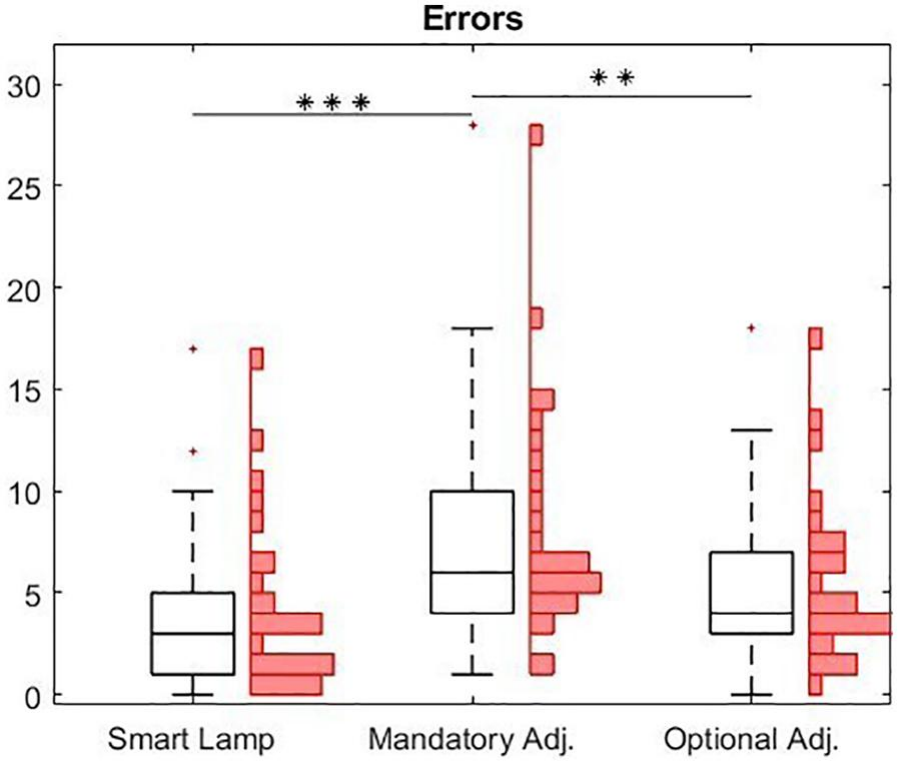
Boxplot of errors arise in the three different conditions. The central line represents the median, while the lower and upper edges of the box correspond to the 25th and 75th percentiles, respectively. In addition to the boxplots, a histogram is provide to illustrate the absolute distribution of error counts for each participant (**p < 0.01; ***p < 0.001).

All errors across the twelve trials were summed for each participant. Errors were defined as incorrect size or shape of the LEGO® bricks, as well as incorrect colors. This allowed the total number of errors per condition to be calculated for each participant. The boxplots representing the total errors per participant for each condition are shown in Figure 5. Additionally, the overall number of errors across all participants was calculated for each condition.

In total, the trials conducted using the smart lamp resulted in the fewest errors, with 111 errors (individual mean: 3.5; SD: 4). In contrast, during the trials with the manual lamp, where participants adjusted the light only when they deemed it necessary, 156 errors (individual mean: 4.9; SD: 3.9) were recorded. The condition requiring participants to manually adjust the lamp once per model led to the highest total error count, with 243 errors (individual mean: 7.6; SD: 5.5) across all participants. A repeated measures ANOVA with a Greenhouse-Geisser correction determined that the mean error rates showed a statistically significant difference between conditions [*F*(1.838, 53.307) = 11.146, *p* < .001, *η*^2^ = 0.278]. A Bonferroni-corrected *post-hoc* test revealed a significantly lower error rate (*p* < .001) when using the smart lamp compared to the manual lamp (mandatory adjustment) [*M*_Diff_ = −4, 95% CI (−6.491, −1.509)].

When examining the effective processing time of participants, defined as the time actively spent searching for the appropriate LEGO® bricks a repeated measures ANOVA with a Greenhouse-Geisser correction determined that mean processing times showed a statistically significant difference between conditions [*F*(1.99, 53.98) = 6,44, *p* = .003, *η*^2^ = 0.18].

**Figure 6:**
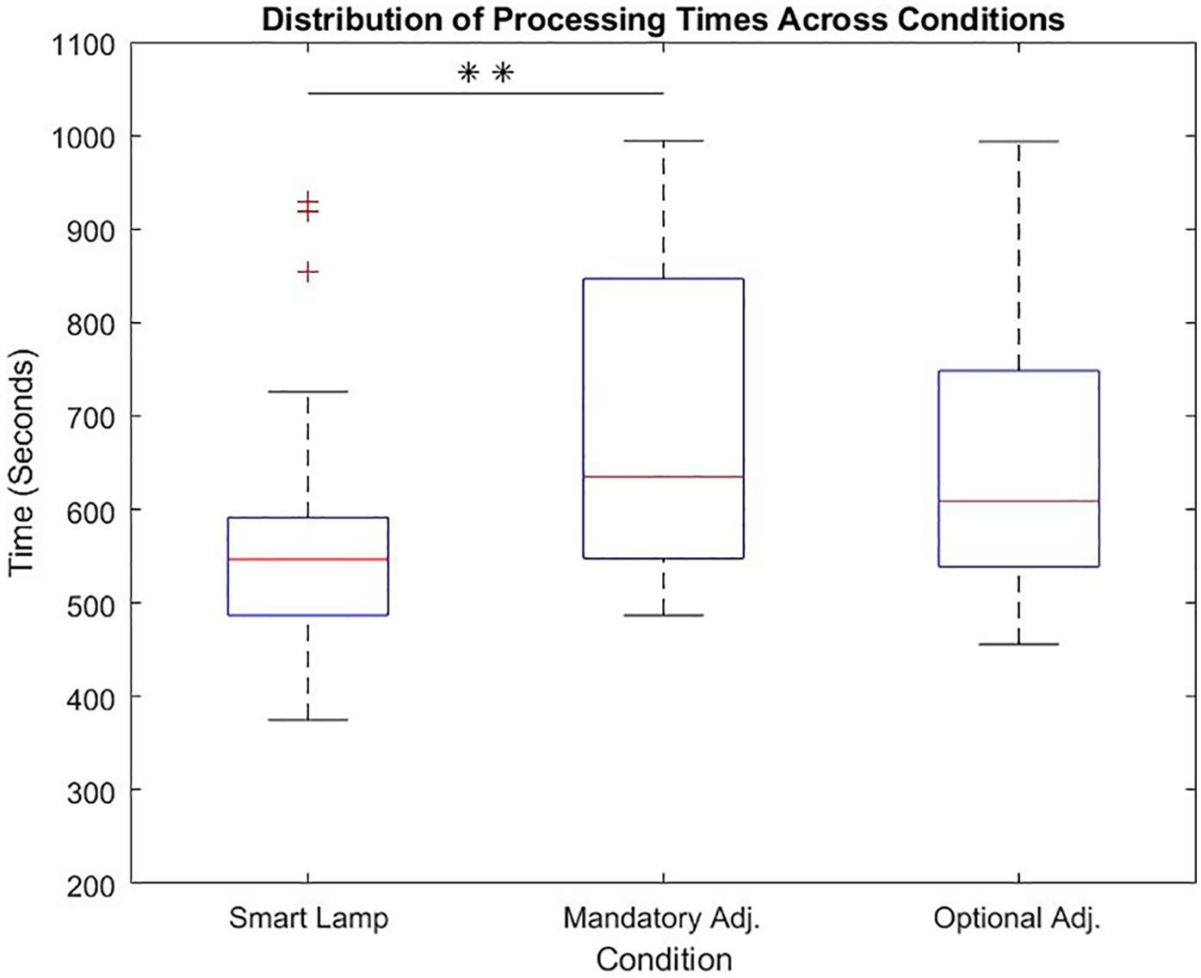
Distribution for processing time across all three conditions (smart lamp, mandatory adjustment, optional adjustment) with significant differences between smart lamp and mandatory adjustment.

A Bonferroni-corrected *post-hoc* test revealed a significantly shorter processing time (*p* = .005) when using the smart lamp compared to the manual lamp (mandatory adjustment) [*M*_Diff_ = −114.41, 95% CI (−198.69, −30.13)]. Comparing the processing times between the smart lamp and the manual lamp (optional adjustment), the Bonferroni-corrected *post-hoc* test revealed no significant (*p* = 0.061) [*M*_Diff_ = −80.124, 95% CI (−163.17, 2.93)]. The corresponding boxplots are presented in Figure 6.

## 4 Discussion

### 4.1 Objective and subjective workload

The findings of this study support Hypothesis 1 that smart surgical lighting reduces cognitive workload compared to manually controlled lighting systems. This was evident in both subjective and objective measures, providing a comprehensive assessment of cognitive demand and its impact on user performance.

The NASA-TLX questionnaire revealed consistently lower scores across all workload categories when the smart surgical lamp was used. Specifically, the “mental demand” was significantly lower in the smart lamp condition compared to both manual conditions—mandatory adjustment and optional adjustment. Similarly, “temporal demand” scores were lowest for the smart lamp, showing significant differences compared to the manual conditions for mandatory and optional adjustments, respectively. These results indicate that the *SmartOT* smart lighting system alleviates mental and temporal strain associated with surgical lighting adjustments.

The effort required to interact with the lighting system was also reduced in the smart lamp condition compared to manual adjustments. Moreover, participants reported significantly lower frustration levels when using the smart lamp, highlighting its potential to enhance user satisfaction and reduce stress during surgical procedures.

These findings align with earlier studies emphasizing the impact of ergonomic optimization in surgical environments (5). Automation in the surgical envionment reduces physical and attentional demands, thereby freeing cognitive resources for primary tasks—a phenomenon well-documented in applied cognitive research (21).

The analysis of EEG data provided an objective assessment of cognitive workload, focusing on the P300 component—a well-established marker of cognitive processing (22, 23). The smart lamp condition yielded significantly higher P300 amplitudes at the Fz electrode (compared to the manual lamp with mandatory adjustments. This suggests improved cognitive efficiency, as higher P300 amplitudes are typically associated with enhanced attention and reduced cognitive load (22). However, no significant differences were observed in the latencies of the P300 component across conditions, indicating that the timing of cognitive processing was unaffected.

This supports theories suggesting that excessive secondary task demands—such as manual equipment adjustment—can reduce attentional capacity for core tasks. By eliminating this interference, smart lighting likely enabled better allocation of cognitive resources (24).

These results suggest that the integration of smart lighting systems in surgical environments can significantly reduce cognitive workload by minimizing the mental, temporal, and physical demands associated with manual adjustments. The improved P300 amplitudes further support the notion that the smart lamp allows participants to allocate cognitive resources more effectively, potentially enhancing task performance and decision-making during surgery.

The work of Mühlenbrock et al. is thus validated by these findings. The consistency between the objective and subjective results of this study also indicates that the methodology used was effective in objectively assessing workplace load (5). A feasibility study previously conducted at our institution (9), as well as the works of Allison and Raabe, which demonstrated the successful objective assessment of workplace load using an Oddball experiment, are further validated by this study (25, 26). Additionally, the works of Pieper and Gibson, which described the consistency between the results of the objective measurement of the workload via EEG and the subject measurement via the NASA-TLX questionnaire (27, 28).

### 4.2 Error rate and processing time

The number of errors was quantified per participant across 12 trials. This included errors in size, shape, and color of each LEGO® brick. Results indicated that the smart lamp condition yielded the fewest errors. While the manual lamp with mandatory adjustments yielded the highest error count. These results suggest that the *SmartOT* adaptive lighting system enhances task accuracy, possibly by reducing visual strain and improving perceptual clarity.

These much higher error rates for the manual lamp conditions may be explained by higher cognitive loads that come with the management of lighting adjustments. Especially, the condition of mandatory adjustment, where participants are enforced to actively change the lighting settings for every model might have brought extra cognitive load that interfered with the performance of the task. This also corresponds to the results of other studies indicating that higher task complexity and more manual interventions result in higher error rates (29).

The effective processing time—defined as the time participants actively spent searching for the appropriate LEGO® bricks—was significantly shorter under the smart lamp condition compared to the manual lamp with mandatory adjustments. This may indicate that adaptive lighting provided by the smart lamp facilitates quicker identification and selection of task-relevant items by improving visual contrast and reducing the need to make manual lighting adjustments.

Although the difference in processing time between the smart lamp and the manual lamp with optional adjustments did not reach significance, a tendency for shorter processing times with the smart lamp was observed. This could indicate that in situations where optional adjustments are permitted but not explicitly required, there may be some benefit from the smart lamp in terms of reduced cognitive load associated with monitoring and adjusting lighting conditions.

These findings strongly support Hypothesis 2, indicating that the error rate was considerably lower under the smart lighting system compared with both manual conditions. This finding further provides support for the efficacy of adaptive lighting in minimizing task errors, likely by reducing cognitive load and providing optimal visual conditions.

Hypothesis 3 is supported because participants in the smart lighting system condition completed tasks more quickly than participants in the manual lamp with mandatory adjustments condition. The comparison with the optional adjustment condition did not reach significance, but the tendency observed does suggest that the smart lighting system may also have contributed to increased efficiency for less demanding manual adjustment scenarios. This interpretation is consistent with the findings of Knulst et al., who emphasize that future improvements in surgical lighting should focus on minimizing both the frequency and the physical effort required for manual repositioning of luminaires—issues that automated lighting systems are specifically designed to address (2).

The present study employed both the EEG recordings and the NASA-TLX questionnaire as concurrent objective and subjective measures of cognitive workload, respectively, in simulated surgical tasks. The multimodal approach contributes a holistic understanding of task-evoked mental effort, combining the physiological with the self-reported experiences. The P300 component based on EEG is a well-established neurophysiological indicator of attention allocation and cognitive resource deployment, particularly under Oddball paradigms (24, 25). The NASA-TLX, by contrast, is a well-validated survey that has been used widely in applied cognitive workload research and provides a quick and interpretable multiple workload dimension measurement (15).

There are other assessment methods, however, which can be used to make additional contributions in future research. One such possible alternative is electrodermal activity (EDA), which reflects sympathetic nervous system activity and has been used widely as an indicator of emotional arousal and stress. EDA recordings are non-invasive, continuous, and relatively easy to use in real-world settings, e.g., the surgical room. Studies have recently proved the usability of using EDA to assess real-time stress responses among surgeons (30).

Relative to EEG, EDA has the advantages of setup simplicity and ecological validity, especially for the tracking of short-term arousal under dynamic surgery conditions. Nevertheless, EDA does not possess the temporal specificity and cognitive granularity of EEG and therefore is less capable of differentiating between diverse cognitive demands (e.g., attention vs. decision-making) (23). EEG, on the other hand, allows more detailed information about underlying neurocognitive processes through time-locked event-related potentials such as the P300 (25).

### 4.3 Limitations

One limitation of this study is the artificial nature of the experimental task, which does not fully replicate the complexity and high-stakes environment of a real surgical procedure. The participants, who had no surgical experience, may have spent less time adjusting the lamps in the study compared to actual surgeries, as the task lacked the urgency and criticality inherent to real-world scenarios. Additionally, the study was not blinded, which could introduce biases in participant behavior and data interpretation. Future research should aim to validate these findings in more realistic clinical settings to better understand the practical implications of smart surgical lighting.

Additionally, while the P300 component provided valuable insights into cognitive workload, further exploration of other EEG mechanisms could offer a more detailed understanding of the neural mechanisms underlying these effects. Incorporating additional measures of biosignals, such as heart rate variability, electrodermal activity or eye-tracking, could also provide a more comprehensive evaluation of cognitive and physical demands.

Moreover, novel variants of mobile EEG systems, such as the cEEGrid, which has already been validated for workload measurement (31), could contribute in future studies to further enhancing mobility. Through even simpler setup and usage, these systems could enable the realization of a more realistic clinical scenario or potentially allow testing in real-world environments. Generalizability of the results is limited due to the specific conditions and participant sample used, highlighting the need for studies in diverse and practical settings.

The risk of contamination through manual control of SLS, which has already been identified as a problem of current SLS systems, is addressed by smart lighting concepts such as the *SmartOT* system. However, this aspect was not further investigated in the present study. Future studies should examine this issue in greater detail. Future studies should examine this issue in greater detail, potentially exploring solutions that integrate automatic controls to reduce contamination risks. Strengths of this study include the introduction of novel smart lighting technology and the integration of EEG to objectively assessing cognitive load, providing a foundation for future research.

## 5 Conclusion

This work demonstrates that using the *SmartOT* prototype significantly reduce errors in a task where participants were required to correctly identify and retrieve LEGO® bricks under conditions simulating a surgical scenario. In contrast, a conventional manually adjustable surgical lamp led to more frequent errors and increased processing time in the mandatory adjustment condition. Additionally, the workload was found to be significantly lower when the *SmartOT* lamp was used, as evidenced by significantly lower NASA-TLX scores for mental demand, temporal demand, effort, and frustration. This was confirmed subjectively using the NASA-TLX questionnaire and objectively using EEG measurements combined with a passive oddball paradigm which revealed a significant difference in the amplitudes of the P300. The latencies did not show significant differences between conditions.

The findings provide preliminary evidence that implementing smart surgical lighting like the *SmartOT* System can enhance patient safety not only directly, by reducing errors and a shorter processing time, but also indirectly, by decreasing the workload of surgeons. Future studies that are more closely aligned with actual surgical conditions and involve medical professionals are needed to further validate these results.

## Data Availability

The raw data supporting the conclusions of this article will be made available by the authors, without undue reservation.
